# Small Changes, Big Impact: Exposure to Air Pollution and Reduced Lung Function in Children

**DOI:** 10.1289/ehp.121-A341

**Published:** 2013-12-01

**Authors:** Julia R. Barrett

**Affiliations:** Julia R. Barrett, MS, ELS, a Madison, WI–based science writer and editor, has written for EHP since 1996. She is a member of the National Association of Science Writers and the Board of Editors in the Life Sciences.

Epidemiological studies suggest that outdoor air pollution adversely affects children’s lung function, which may not only harm their health now but also increase their susceptibility to respiratory and cardiovascular disease in adulthood.^1^^,^^2^^,^^3^^,^^4^ Exposure to air pollutants in infancy is suspected to alter lung development, with potential long-term consequences.^5^ However, a new study in *EHP* encompassing exposures at birth and at school age found that decreases in lung function were associated only with recent exposure.^6^

Researchers analyzed data from five European cohort studies, which assessed exposure using a standardized method.^7^ The five cohort studies, conducted in Sweden, northern and southern Germany, the United Kingdom, and the Netherlands, were intended in part to study the development of asthma and allergies.

The studies measured lung function of participants at age 6 or 8 years and used questionnaires to collect information about the children, their homes and families, and numerous factors touching on lung health. Land-use regression models provided estimates of annual average concentrations of specific air pollutants at the children’s home addresses at two time points: when they were newborns (between 1994 and 1999) and when they underwent lung function testing 6 or 8 years later. These models were based on actual monitoring that occurred in 2008–2010, when the participants were adolescents.

The analyses of 5,921 children revealed small but significant associations between decreased lung function and higher estimated levels of pollution at the children’s home address at age 6–8, but not at their address as a newborn. This finding suggests that the recent exposures were the more critical to current lung function.

Based on two earlier studies that investigated exposure at more than one time point,^8^^,^^9^ an association between later lung function and early-life exposure might have been expected, says Ulrike Gehring, the study’s lead author and an assistant professor at the Institute for Risk Assessment Sciences, Utrecht University. However, she also highlights other evidence that the effects of air pollution on children’s lung function may be reversible.^10^^,^^11^

**Figure:**
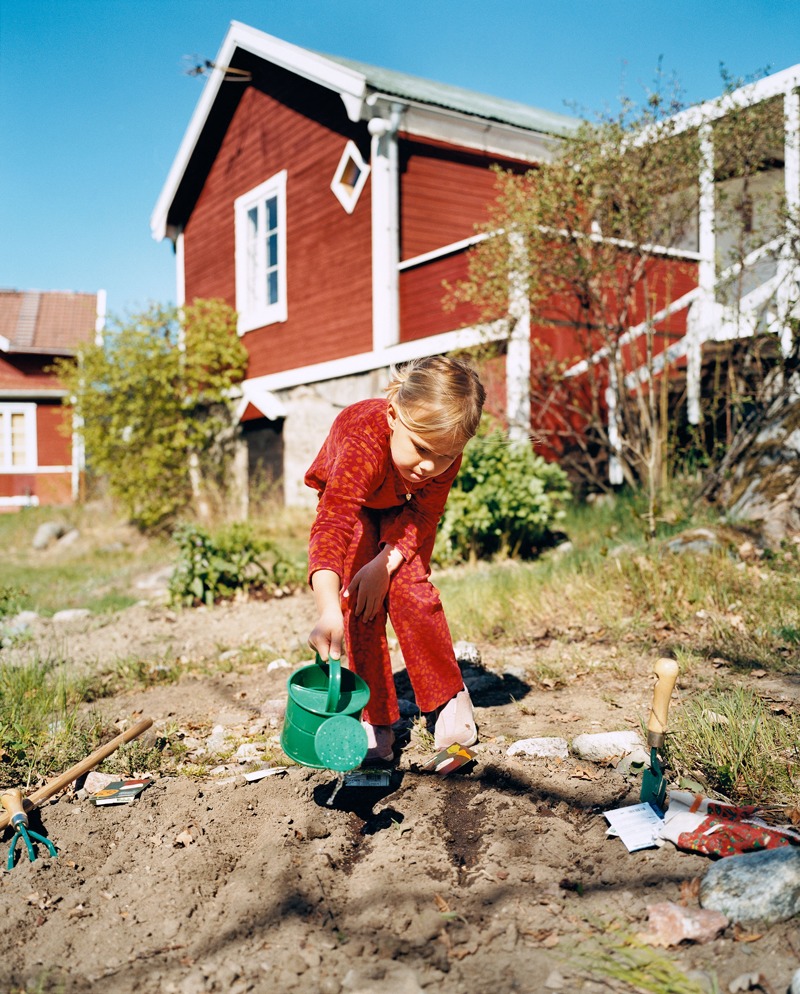
Small reductions in lung function like those reported among five cohorts of European children could increase the risk of asthma and chronic obstructive pulmonary disease later in life. © Johner Images/Alamy

Although small, the changes reported in the current study could have a disproportionate impact on public health, says Gehring. “These small changes result in a considerable increase in the number of children with a clinically low lung function. Further, a lower lung function in childhood may predispose children for asthma and chronic obstructive pulmonary disease later in life,” she says.

Because exposure estimates were limited to children’s residences, they did not capture exposures that may have occurred elsewhere, such as at schools or child-care centers. The current investigation also could not pinpoint the specific effects of individual pollutants, nor could it reveal the mechanisms by which damage might occur.^6^

Nevertheless, the study solidly supports an association between air pollution and adverse effects on children’s lung function, according to Jonathan Grigg, a professor of pediatric respiratory and environmental medicine at the London School of Medicine and Dentistry, who was not involved in the study.

“Clearly, a big strength is that [the researchers] put a lot of effort into harmonizing their land-use regression model, which I think means that these data are robust—as robust as we can have, given what epidemiology can do,” says Grigg. “However, we’re sort of at the limit of what we can do with epidemiology, using big numbers, large-scale studies, modeling exposure, and trying to look at the independent effects of pollutants.”

Previous studies have suggested that oxidative stress and inflammation are the primary mechanisms by which ambient air pollution induces adverse health effects. These mechanisms may also be relevant to lung function, but toxicological evidence is sparse, and very limited animal data exist. Smaller studies in which individual exposure is measured in more detail and linked with lung function may be the next phase in this area of research, according to Grigg. He says animal models could demonstrate causality between exposure and effect, with the caveats that lung growth differs among species, and such studies are expensive and complex.

Gehring says children in the current analysis are still being tracked. Most of the cohort studies have followed up at ages 10–12 and 16 years, and the teams plan to follow the children into adulthood. This will allow them to explore questions such as whether deficits in lung function in adolescence set the stage for reduced lung function in adulthood.^12^
